# Validating Acute Coronary Syndrome ICD Codes as Training Labels for Clinical AI

**DOI:** 10.21203/rs.3.rs-11156040/v1

**Published:** 2026-10-19

**Authors:** Daniel K. Amponsah, Gabrielle Bunney, Kate Miller, Ariadna Garcia, Anna Graber-Naidich, Keejeong Ryu, Himani Darji, Marilyn Ndukwe, Valerie J.F. McDonald, William M. Mehring, Jada Henderson, David Lei, Marium Rizvi, Adenine Cembellin-Kao, Sylvia D. Kreibig, Celina M. Yong, William F. Fearon, Maame Yaa A.B. Yiadom

## Abstract

Artificial intelligence models may improve emergency department (ED) screening for acute coronary syndrome (ACS), but such models often rely on International Classification of Diseases (ICD) codes as training labels, whose accuracy in contemporary ED populations is uncertain. In this 10-year retrospective study of 619,599 adult ED encounters, we compared ICD-coded ACS against a chart-reviewed reference standard defined as the treating team’s documented diagnosis. Sensitivity, specificity, and positive predictive value were 94.4%, 99.7%, and 72.4%, respectively. Disagreement occurred in 0.31% of encounters. Among false positives (n=1,647), the leading reason was ACS suspected but later ruled out (80.3%); among false negatives (n=257), coder omission of documented ACS (86.0%). ACS ICD codes are suitable training labels for screening models where missing ACS is more consequential than over-identification, and the observed error patterns are systematic and interpretable rather than random.

